# A randomized controlled trial with a Canadian electronic pill dispenser used to measure and improve medication adherence in patients with schizophrenia

**DOI:** 10.3389/fphar.2013.00100

**Published:** 2013-08-09

**Authors:** Emmanuel Stip, Philippe D. Vincent, Juliette Sablier, Catherine Guevremont, Simon Zhornitsky, Constantin Tranulis

**Affiliations:** ^1^Centre de Recherche Fernand-Séguin, Institut Universitaire en Santé Mentale de MontréalMontreal, QC, Canada; ^2^Department of Psychiatry, Faculty of Medicine, Université de MontréalMontreal, QC, Canada; ^3^Department of Psychiatry, Institut Universitaire en Santé Mentale de MontréalMontreal, QC, Canada; ^4^Faculty of Pharmacy, Université de MontréalMontreal, QC, Canada; ^5^Department of Pharmacy, Institut Universitaire en Santé Mentale de MontréalMontreal, QC, Canada; ^6^Cognitive Neuroscience Centre, UMR 5229 of CNRS, Psychology Institute, Université Lyon 2Lyon, France

**Keywords:** adherence, randomized, DoPill^®^, MEMS^®^, pill dispenser, schizophrenia

## Abstract

**Objective:** Medication adherence is extremely important in preventing relapse and lowering symptoms in schizophrenic patients. However, estimates show that nearly half of these patients have poor adherence. The Brief Adherence Rating Scale (BARS) seems to be the most reliable tool assessing adherence in schizophrenia and shows that the antipsychotic adherence ratio (AAR) is about 49.5% in schizophrenia. The aim of the study was to test if an electronic pill dispenser named DoPill^®^ improved AAR of schizophrenic patients. Furthermore, we compared AAR obtained by the DoPill^®^ and the BARS, in order to verify whether the DoPill^®^ provides reliable assessment of medication adherence.

**Methods:** The DoPill^®^ is a smart pill dispenser that beeps and flashes at the appropriate time of the day. Each of its 28 compartments is covered by a plastic lamina that, when taken off, sends a signal to the pharmacist. Patients were randomized to the DoPill^®^ or treatment as usual groups for 6 weeks. The BARS was used as a reference measure.

**Results:** Forty-six percent of patients were deemed to be non-adherent with antipsychotic medication. The mean AAR was 67% after 6 weeks. DoPill^®^ recorded better AAR than some of those found in the literature and were lower than the BARS estimate we found.

**Conclusion:** These results suggest that DoPill^®^ is a valid tool that provides more reliable and objective data for the clinician about their patient’s adherence, than existing assessment tools like the BARS. Furthermore, the device may help patients successfully manage their medication regimen.

## INTRODUCTION

The first decade of illness in schizophrenic patients is often characterized by repeated episodes of psychosis with varying levels of remission between episodes and increased disability following each episode, especially when untreated by antipsychotic medication (Ciompi, 1980; Wyatt, 1991; Tandon et al., 2009; Malla et al., 2011). Despite the established benefits of antipsychotic therapy in schizophrenia, all three components of adherence are affected: initiation, implementation, and discontinuation (Vrijens et al., 2012). Data from 2,588 first-episode psychosis (FEP) patients revealed both initiation and discontinuation non-adherence: only 58% collected their prescription during the first 30 days of hospital discharge and only 46% continued their initial treatment for 30 days or longer (Tiihonen et al., 2011). The implementation component of non-adherence was studied in a semi-quantitative review of the literature which found that 49.5% of patients with schizophrenia were deemed non-adherent according to the criteria: “taking medications as prescribed at least 75% of the time” and 41.2% were deemed non-adherent according to the criteria: “regularly taking medications as prescribed” (Lacro et al., 2002). In practical terms, there is evidence that non-adherence doubles the rate of readmission (Valenstein et al., 2002, 2004; Morken et al., 2008) which is about 4–6% according to Valenstein et al. (2006). However, recovery and long-term outcomes were not correlated with adherence in a recent well-designed discontinuation FEP study (Wunderink et al., 2009). Moreover, administering antipsychotics every other day was not associated with higher relapse rate or lower side effects in a randomized controlled trial on 35 stable patients (Remington et al., 2011), suggesting that in some patients sub-optimal implementation of prescriptions might represent a form of lay dosage optimization.

Clinicians can increase medication adherence in schizophrenia patients via prescription of long-acting injectable antipsychotics or non-pharmacological interventions as cognitive behavioral therapy, psychoeducation, family intervention, and motivational approaches (Zhornitsky and Stip, 2012). Recently, technologically sophisticated electronic reminder devices and electronic pill dispensers have been developed (Diaz et al., 2001). Among these, the Medication Events Monitoring System (MEMS^®^) has attracted the most interest in the literature (Byerly et al., 2005; Remington, 2008; Acosta et al., 2009; Lee et al., 2011). It consists of a cap containing an electronic chip that records openings of the bottle; the cap contains a liquid crystal display (LCD) screen that shows the number of times the bottle has been opened in the day and hours since its last opening. Thus, it is imprecise because it can be opened more or less often than necessary. Also, its main purpose is only to monitor rather than to improve management of adherence. Studies in schizophrenia that have quantified implementation of drug regimen with MEMS^®^ have found that between 41.2 and 59% of patients take >70% of it (Byerly et al., 2007, 2008; Remington et al., 2007; Acosta et al., 2009).

There is a lack of objective tools to precisely and specifically assess the antipsychotic adherence ratio (AAR = number of pills taken × 100/number of pills prescribed, a quantification of implementation). In their systematic review of assessments tools of antipsychotic adherence, Velligan et al. (2006) highlight that 77% of the methods reported in the literature are self-reported and indirect, whereas only 23% reported using an objective and direct assessment like counting pills, measuring blood or urine concentration, and electronic monitoring. Although it provides indirect and self-rated measures, the Brief Adherence Rating Scale (BARS) is a reliable tool to detect sub-optimal implementation of the dosing regimen in schizophrenic patients. It has demonstrated good reliability, sensitivity, and specificity relative to MEMS^®^ (Byerly et al., 2008). According to this scale, the AAR is about 49.5% in schizophrenia. Yet, self-evaluation may over-estimate the real AAR (Byerly et al., 2007).

In summary, non-adherence is a core problem in schizophrenia, but there is no validated tool to quantify implementation of drug regimen in real time, and there is a lack of effective tool to improve management of adherence in these patients. Recently, an electronic, smart, 7-days pill dispenser named DoPill^®^ was developed by Domedic in Quebec to simultaneously quantify and help patients with implementation. When we learned its existence, we immediately wondered if its potential could help our patients suffering from schizophrenia.

In the present randomized trial, we used the DoPill^®^ to provide estimates of AAR in FEP patients as well as to quantify implementation in real time. We hypothesized that this smart pill dispenser would provide objective and direct measures of implementation comparable with those found with the BARS, and that the reminders would help patients improve their management implementation of the drug regimen.

## MATERIALS AND METHODS

### PARTICIPANTS

Participants with schizophrenia according to the fourth edition of Diagnostic and Statistical Manual of Mental Disorders IV (DSM-IV) criteria were recruited at the Institut universitaire en santé mentale de Montréal between January 2008 and September 2010. Patients were randomized in two groups. The experimental group used the DoPill^®^ during 6 weeks, and the control group continued to take their treatment as usual (TAU; Sablier et al., 2009). The study was approved by the Institut universitaire en santé mentale de Montréal’s ethics committee and a signed informed consent obtained from all participants prior to study entry.

### THE DoPill^®^

**FIGURE 1:**
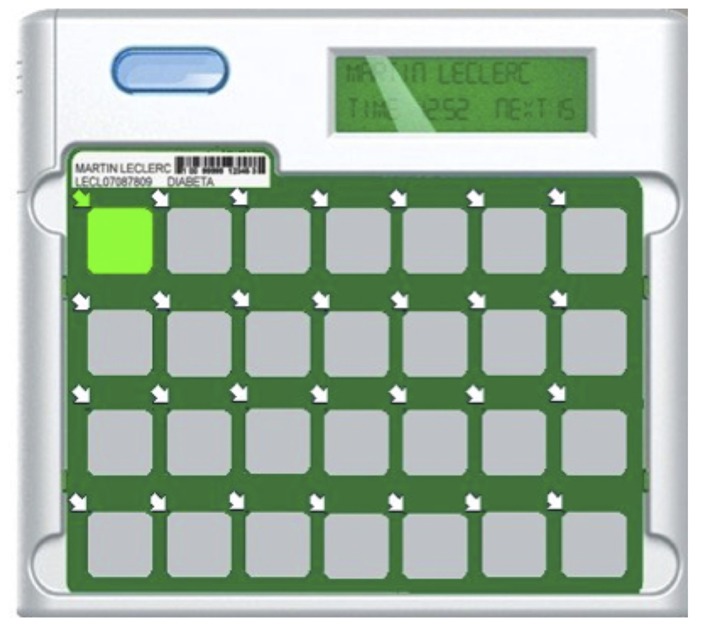
**DoPill^®^ – an intelligent electronic pill dispenser**.

DoPill^®^ is an electronic dispenser with 28 compartments covered by a dynamic membrane which allows events detection (**Figure 1**). Each compartment can contain multiple pills. The patient’s pharmacist is responsible for programming the device, doing the usual safety checks, and dispending the medication as prescribed. Visual and sound alarms alert the user when it’s time to take medication and the compartment from which it must be taken. Finally, sensors record the time of opening and upload the data to a secure server on Internet which calculates an adherence ratio (pill taken/pills given), and where clinicians can consult the adherence in real time. Ten prototypes were provided free of charge by the manufacturer.

### PROCEDURE

Study protocol consisted of three visits: one baseline, one at 6 weeks, and one at 8 weeks. Patients were evaluated with the BARS and the positive and negative syndrome scale for schizophrenia (PANSS) to assess DoPill’s^®^ impact on psychiatric symptoms. The patient and clinician-rated measure of drug adherence by the BARS is separated in two steps. Initially, the clinician asks the patient how often he tends to forget to take medication. Subsequently, it’s the clinician that indicates the proportion of medication he/she believes that the patient consumes.

### STATISTICAL ANALYSIS

The DoPill^®^ software calculated AAR for all patients, and computed the mean adherence rate of antipsychotics in the experimental group. This result was statistically compared with the BARS in both group assignments. Mean adherence rate was also compared numerally with AAR found in the literature using MEMS^®^. Proportions of patients with <70 and <90% AAR was also computed, and compared numerally with AAR found in the literature using MEMS^®^. The later is adapted from Weiden et al. (2004) who found that under <70% of mean possession ratio (a composite of initiation, implementation, and discontinuation), and <90% persistence, 8.5 and 10.6% more patients are hospitalized over one year, respectively. For the sake of clarity, patients with <70% AAR are considered non-adherent. Cost of non-adherence was estimated by the cost of wasted pills.

Descriptive statistics were used for baseline sociodemographic and clinical characteristics of patients, for cost analysis, and for proportion of patients with <70 and <90% AAR.

The results in the BARS did not follow a normal distribution since most scores fell toward 100% adherence. However, a Brown–Forsythe test allowed us to verify homogeneity of variance. Repeated measures ANOVA, with adherence rate, BARS, group and visit number as variables, were preformed with the non-parametric Scheirer–Ray–Hare test extension of the Kruskal–Wallis test. The level of significance for two-sided hypothesis tests was set at a *P*-value less than 0.05. The SPSS 17.0 (Chicago, IL, USA) statistical software was used for all analyses.

## RESULTS

### PARTICIPANTS

Completion of the study was 26/36 in the experimental group, and 21/28 in the TAU group. Group sizes differ because of chance in the randomization process, and the sample size is modest because of time restrictions related to academia and human resources. Dropout reasons were varied: in the experimental group, the DoPill^®^ sometimes had technical problems and patients dropped out, some patients changed their minds when they were randomized to TAU, and others were hospitalized.

Baseline sociodemographic and clinical data are shown in **Table 1**. No significant differences were noted for age, education, illness duration or PANSS positive, negative and general scores between patients randomized to the DoPill^®^ and the TAU groups. All were outpatients suffering from moderate illness. Although the patients were recruited from a FEP program, mean age and duration of illness were 38 and 11 years, respectively, in the DoPill^®^ group, and 35 and 9 years, respectively, in the TAU group. In the DoPill^®^ group, antipsychotic prescriptions were as follows (six patients were on antipsychotic polypharmacy): olanzapine (*n* = 7), clozapine (*n* = 7), quetiapine (*n* = 4), ziprasidone (*n* = 3), risperidone (*n* = 2), haloperidol (*n* = 2), perphenazine (*n* = 2), paliperidone (*n* = 1), zuclopenthixol (*n* = 1). In the TAU group, antipsychotic prescriptions were as follows (eight patients were on antipsychotic polypharmacy): olanzapine (*n* = 8), clozapine (*n* = 6), risperidone (*n* = 5), quetiapine (*n* = 3), ziprasidone (*n* = 2), haloperidol (*n* = 2), perphenazine (*n* = 2), aripiprazole (*n* = 1), fluphenazine (*n* = 1). Concomitant medications were allowed and dispensed in the DoPill^®^ by the pharmacist as usual.

**Table 1:** Baseline sociodemographic and clinical characteristics of randomized patients.

	DoPill^®^(*n* = 26)	TAU (*n* = 21)	Statistics
Age	38 (10)	35 (9)	*t* = 1.32; *p* = 0.19
Education	12 (3)	11 (3)	*t* = 0.98; *p* = 0.33
Illness duration	11 (10)	9 (8)	*t* = 0.76; *p* = 0.45
PANSS positive	16 (5)	17 (4)	*t* = –0.69; *p* = 0.5
PANSS negative	19 (5)	18 (5)	*t* = 0.58; *p* = 0.57
PANSS general	40 (7)	39 (5)	*t* = 0.77; *p* = 0.45
**Gender**
Male	62%	76%	
Female	38%	24%	
**Diagnosis**
Schizophrenia	71%	84%	
Other^*^	29%	16%	

* Schizoaffective or schizophreniform disorder.

### MEASURING ADHERENCE WITH BARS

The BARS results are shown in **Table 2**. The repeated measures ANOVA failed to reveal an interaction between the factors “group” and “time” for the BARS patient self-report adherence rate (*P* = 0.73). On the other hand, a repeated measures ANOVA revealed an interaction between group and visit for the BARS clinician rating (*P* = 0.01). Contrast tests allowed us to verify that this difference is present between both baseline and visits 1 and visits 2.

**Table 2:** Adherence ratings across visits (BARS).

	Baseline, mean (SD)	Visit 1, mean (SD)	Visit 2, mean (SD)	*P*-value^*^
**Clinician rating (DoPill^®^)**
Adherent (*n* = 12)	92.6 (7.6)	98.3 (3)	96.3 (3.5)	*P *= 0.01
Non-adherent (*n* = 8)	95.9 (3)	86.4 (11.1)	93.3 (9.5)	
**Self-report (DoPill^®^)**
Adherent (*n* = 13)	95.9 (8.1)	96.2 (11.1)	99 (2.5)	*P *= 0.73
Non-adherent (*n* = 11)	93.3 (11.2)	95.2 (6.9)	94.9 (5.2)	

* Interaction (visit × group).

### MEDICATION ADHERENCE WITH DoPill^®^ VERSUS MEMS^®^

**FIGURE 2:**
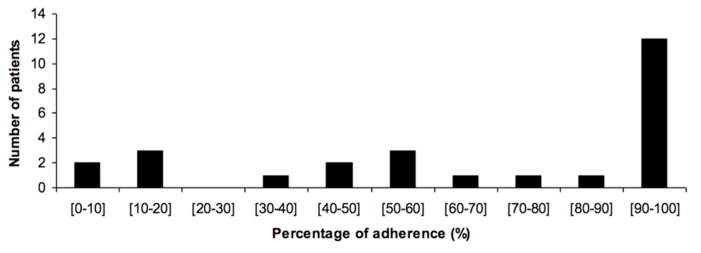
**Adherence to treatment as recorded by the DoPill^®^**.

Adherence rates recorded by DoPill^®^ for each patient are presented in **Figure 2**. In this sample, the mean AAR recorded by DoPill^®^ over the 6 weeks of use was 66.6% [secure digital (SD) 35.1]. The proportion of patients with <70 and <90% AAR was 46 and 54%, respectively, which was consistent with 41.2–59% found with MEMS^®^ in the literature.

#### Potential heterogeneity

In the data set, extracted for each patient, we noticed that some patients did not take all the medication prescribed by the psychiatrist and choose or selected only some of the pills from the dispenser. As illustrated in **Table 3**, we can see that for instance in subject EET-20 venlafaxine was 38% taken versus 43% for procyclidine. This can also be explained if the patient forgot most often morning drugs but not evening ones because of environmental or time factors.

**Table 3:** Estimation of non-adherence costs in our sample of schizophrenia patients (*N* = 17). Example of two patients from the “Enriched Environment withTechnology” (EET) group.

Patient #	Medication	AAR (%)	Price per unit ($/u)	Unused costs per year (CAD)
EET-01 (AAR = 96.08%)	COGENTIN 2 mg	93.33	0.23	$ 35.62
	SEROQUEL XR 400 mg	100	4.56	
	ZELDOX 40 mg	94.74	1.94	
	ZELDOX 60 mg	92.31	1.94	
	SEROQUEL XR 200 mg	100	2.46	
EET-20 (AAR = 42%)	PMS-PROCYCLIDINE 2.5 mg	42.86	0.24	$ 1,638.43
	HALOPERIDOL 2 mg	42.86	0.12	
	HALOPERIDOL 5 mg	42.86	0.18	
	GLUCOPHAGE 500 mg	40.82	0.39	
	VENLAFAXINE XR 150 mg	38.78	2.24	
	GEN-CLOZAPINE 50 mg	42.86	0.7	
	GEN-CLOZAPINE 200 mg	42.86	1.9	
Total (*N = *17)		63.51		$ 9,823.83

*AAR, antipsychotic adherence ratio.*

#### Cost

Cost related to non-adherence (pills actually not taken), was estimated at 15024.68 CAN $/year for the 26 patients we followed. See **Table 3** for calculations for two subjects.

## DISCUSSION

This randomized trial revealed that the DoPill^®^ is a well-designed, technologically sophisticated, and highly functional electronic pill dispenser. The device provided estimates of implementation in FEP patients from the proportion of doses taken on time, which were in accordance with those found in the literature (Diaz et al., 2001; Lacro et al., 2002; Byerly et al., 2005; Remington, 2008; Lee et al., 2011). By contrast, BARS scores were overly optimistic (86–99%) for adherent and non-adherent patients across visits. Previous research has shown a high degree of concordance between BARS and MEMS^®^ over a period of 6 months (Byerly et al., 2008). However, in our study the scale was filled by a nurse, an ergotherapist or a specialized educator, but not by the psychiatrist of the patients. Our results suggest that the BARS is more reliable if filled by the psychiatrist of the patient. Recently, another group from Montreal evaluated how much agreement exists between self-reports of adherence to antipsychotic medication and objective or derived measures of adherence in FEP (Lepage et al., 2010). Adherence was measured in 81 FEP subjects on a monthly basis by reports from patients, clinicians, family, and pill counting. Adherence (74%) as measured by patient report, pill count, and clinician reports were in good agreement with each other and all of these measures were highly correlated to consensus adherence. The authors showed that patient or clinician reports gave a reasonable estimate of medication adherence in FEP, and that introducing pill counting was a better accurate measurement. We think that this high score of adherence is probably optimistic.

Data from DoPill^®^ revealed a mean adherence rate of 67%, which is highly consistent with previous data on MEMS^®^ (Byerly et al., 2005; Remington, 2008; Acosta et al., 2009; Lee et al., 2011). Additionally, when treated as a dichotomous variable (<70% criteria), 46% of patients were found to be non-adherent using the device. At outset, it was reasonable to expect that non-adherence rates obtained with DoPill^®^ would be substantially lower over those obtained with MEMS^®^ because the former employs electronic cues designed to counter forgetfulness. Although we did not compare them directly, we believe they are technically comparable for measurement because they both record the opening of the pill compartments. Despite the cues, we found comparable rates of non-adherence using both devices at the <70% threshold. Looking at the raw scores in **Table 2**, we can see that most of adherent DoPill^®^ patients are *ultra-adherent* (i.e., 98–100%), suggesting that the device may maximize adherence in those already adherent, while leaving the overall rates of non-adherence (<70% criteria) at similar levels. In support, the BARS clinician ratings showed that adherent patients evidenced significantly greater improvement in adherence, relative to non-adherent patients over the 6 weeks. Taken together, these preliminary findings suggest that there may be a limit on the benefit that electronic aids can have for increasing the implementation of the drug regimen in non-adherent schizophrenia patients. That is, we can maximize it in “responsive” FEP patients, but other interventions are necessary to reach the ~46% of patients who are non-adherent (<70% criteria), despite electronic aids. This is compatible with a model of five patient prototypes of adherence: patients with (1) good adherence for the right reasons, (2) with good adherence for the wrong reasons, (3) with passive adherence, (4) with reluctant and tenuous adherence, and (5) with unwillingness to take medications (Freudenreich and Tranulis, 2009). Indeed, the DoPill^®^ is likely to improve the management of adherence only in the first three prototypes, and to be of little benefit for the later two, who are also probably likely to be completely non-adherent (i.e., not simply forgetful).

To compare the DoPill^®^ with other electronic pill dispensers, we searched the World Wide Web and PubMed. We found that the most popular devices are simple alarm clocks with vibration, and the most sophisticated are alarms that also dispense the medication and can call a caregiver if forgotten. However, MEMS^®^ excluded, there are no other device that integrates the pharmacist in the process, and no other device that were studied in a clinical trial. This professional is the most important to ensure that drug regimens are safe and effective, and it’s critical that he validates the final disposition and identification of drugs before administration. This is a significant strength of the DoPill^®^, because it’s the patient’s pharmacist who prepares and identifies the 7 days medication card, and who sends the data in the patient’s device by Internet. All this is integrated seamlessly in the pharmacy software, with the usual security checks. MEMS^®^ remains the most studied device to monitor adherence, but it does not include reminders, and every dosage of a drug needs a separate device. DoPill^®^ has the potential to replace it, because it includes reminders, monitors opening in real time, can communicate with a provider, and can dispense multiple drugs at a time.

The study has also some limitations. The imbalance between the two groups as well as the low completion rate have to be considered. Results could have been different with a bigger sample size, a fully functional DoPill^®^, and longer duration of the trial. Unfortunately, every trial or study in technology or device development can be confronted with time restrictions to complete the study due to the evolution of the device. For many people who suffer from reduced cognitive and intellectual capacity, the complexity of user interfaces, their logic and configuration appear to be the biggest challenge, but many designers and manufacturers still overlook these problems. Therefore, simplifying user interfaces should be a designer’s and software engineer’s main focus, but this limits clinical trial duration to give the opportunity to developers to test new improved versions.

Universal design is a term which refers to accessible, comprehensible, and intuitive design solutions for all, regardless of age, ability, or status, but also solutions that avoid stigmatization and digital exclusion. In schizophrenia it is crucial. Today more than ever, designers are sensitive to the need for simple and meaningful products, especially considering the complexity and continuously changing nature of digital products and devices. In our trial, the findings and patient feedback were continuously shared with the manufacturer to improve our patient’s safety and satisfaction with the DoPill^®^. It is presently used in clinical practice, because physical and psychological barriers have been reduced, and its user interface is intuitive and decipherable.

## CONCLUSION

Overall, results from this randomized study indicate that DoPill^®^ is a valid tool to quantify adherence. It also has the added advantage of being able to track implementation daily, in ecological conditions, and to send a signal to the pharmacist or another person in case of problem or missed dose. It is also clear with the high costs of non-adherence that a constant monitoring of a patient’s adherence is extremely useful. In FEP patients, reported rates of non-adherence [39% with Coldham et al. (2002); 33% with Kamali et al. (2006)] varied with time and typically increased during months six to twelve (Lepage et al., 2010). We suggest that it would be of value to use DoPill^®^, or any smart electronic pill dispenser with live monitoring, as a standardized objective measure of adherence in clinical trials. A longer trial is needed to confirm this, but in the meantime, these devices can be used to help selected patients to successfully manage their medication regimen.

## Conflict of Interest Statement

The authors declare that the research was conducted in the absence of any commercial or financial relationships that could be construed as a potential conflict of interest.

## Abbreviations

AAR: antipsychotic adherence ratio
BARS: Brief Adherence Rating Scale
DSM-IV: Diagnostic and Statistical Manual of Mental Disorders IV
EET: Enriched Environment with Technology
FEP: first-episode psychosis
LCD: liquid crystal display
MEMS^®^: Medication Events Monitoring System
PANSS: positive and negative syndrome scale for schizophrenia
TAU: treatment as usual group
